# Conidial Morphogenesis and Septin-Mediated Plant Infection Require Smo1, a Ras GTPase-Activating Protein in *Magnaporthe oryzae*

**DOI:** 10.1534/genetics.118.301490

**Published:** 2018-11-16

**Authors:** Michael J. Kershaw, Magdalena Basiewicz, Darren M. Soanes, Xia Yan, Lauren S. Ryder, Michael Csukai, Miriam Oses-Ruiz, Barbara Valent, Nicholas J. Talbot

**Affiliations:** * School of Biosciences, University of Exeter, EX4 4QD, UK; † Biological Sciences, Syngenta, Jeallott’s Hill International Research Centre, Bracknell RG42 6EY, UK; ‡ Department of Plant Pathology, Kansas State University, Manhattan, Kansas 66506

**Keywords:** *Magnaporthe oryzae*, *Pyricularia oryzae*, rice blast, Smo, Ras-Gap, bulked segregant analysis

## Abstract

The pathogenic life cycle of the rice blast fungus *Magnaporthe oryzae* involves a series of morphogenetic changes, essential for its ability to cause disease. The *smo* mutation was identified > 25 years ago, and affects the shape and development of diverse cell types in *M. oryzae*, including conidia, appressoria, and asci. All attempts to clone the *SMO1* gene by map-based cloning or complementation have failed over many years. Here, we report the identification of *SMO1* by a combination of bulk segregant analysis and comparative genome analysis. *SMO1* encodes a GTPase-activating protein, which regulates Ras signaling during infection-related development. Targeted deletion of *SMO1* results in abnormal, nonadherent conidia, impaired in their production of spore tip mucilage. Smo1 mutants also develop smaller appressoria, with a severely reduced capacity to infect rice plants. *SMO1* is necessary for the organization of microtubules and for septin-dependent remodeling of the F-actin cytoskeleton at the appressorium pore. Smo1 physically interacts with components of the Ras2 signaling complex, and a range of other signaling and cytoskeletal components, including the four core septins. *SMO1* is therefore necessary for the regulation of RAS activation required for conidial morphogenesis and septin-mediated plant infection.

*MAGNAPORTHE oryzae* (synonym of *Pyricularia oryzae*) is an ascomycete fungus responsible for rice blast disease (Zhang *et al.* 2016), a devastating plant disease that results in severe losses to the global rice harvest each year. The need for increased rice production to feed the rapidly expanding human population, together with the increasing energy costs of both fungicides and fertilizers, means that there is an urgent need to develop durable rice blast control strategies to be deployed as part of an environmentally sustainable plan for increasing global rice production (Wilson and Talbot 2009; Yan and Talbot 2016).

The rice blast fungus initiates plant infection when a three-celled spore, or conidium, lands and germinates on a leaf surface. Conidia are able to adhere to the hydrophobic leaf surface by means of spore tip mucilage (STM), which is released from a compartment at the tip of the apical cell of the spore. Apical conidial attachment, together with the pyriform shape of the spore, are hydrodynamically favorable for resisting water flow and maintaining attachment to the leaf as the spore germinates (Hamer *et al.* 1988). Typically, a single polarized germ tube emerges from the spore and, after 4–6 hr, the tip of the germ tube swells and then differentiates into a specialized infection cell called an appressorium (Talbot 2003; Ryder and Talbot 2015). In the appressorium, a discrete melanin cell wall layer is essential for the generation of high internal turgor pressure, by facilitating the accumulation of glycerol to very high concentrations (de Jong *et al.* 1997). Penetration of the host cuticle results from the application of turgor as a mechanical force, leading to protrusion of a rigid penetration peg to rupture the leaf cuticle. Repolarization of the appressorium requires septin-mediated F-actin reorganization at the base of the appressorium (Dagdas *et al.* 2012). The fungus invades host cells, colonizing tissue rapidly, which leads to the formation of disease lesions from which the fungus produces large numbers of spores, allowing rapid spread of the disease to neighboring plants (Ou 1985).

The *M. oryzae SMO1* locus was first defined from multiple mutants identified spontaneously or through genetic screens that took place more than 25 years ago (Hamer *et al.* 1989b). One screen aimed to identify factors contributing to appressorium development and another involved the isolation of mutants that were unable to adhere to hydrophobic surfaces, such as Teflon (poly-tetrafluoro-ethylene). All mutants formed aberrantly shaped spores, with no visible axis of symmetry. Wild-type conidia in *M. oryzae*, by contrast, are bilaterally symmetrical and pyriform (tear-drop) shaped. These spore morphology mutants were named Smo and tetrad analysis showed that the phenotype was due to a single gene mutation that defined a new locus, *SMO1*, involved in cell shape determination. Smo1 mutants also developed misshapen asci and affected appressorium morphogenesis (Hamer *et al.* 1989b). The original *smo1* mutants were identified in a weeping lovegrass (*Eragrostis curvula*)-infecting *M. oryzae* strain 4091-5-8 (Hamer *et al.* 1989b), but *smo1* mutants were later isolated and characterized in a rice pathogen of *M. oryzae*, and showed a virulence defect when inoculated on susceptible rice cultivars (Hamer and Givan 1990). The *SMO1* locus was mapped based on the segregation of a dispersed repeated DNA sequence, called MGR586 (Hamer *et al.* 1989a), and shown to be located between two closely linked MGR sequences (Romao and Hamer 1992). However, an exhaustive series of map-based cloning experiments and complementation analysis failed to clone *SMO1*, so that its identity has remained unknown for the last 25 years.

Here, we report the identification of *SMO1* using comparative genome analysis and bulked segregant analysis (BSA) (Michelmore *et al.* 1991) of pooled DNA samples from the progeny of a genetic cross of *M. oryzae* segregating for the Smo1 mutant phenotype. Complementation of the original Smo1 mutants followed by targeted gene deletion confirmed the identity of *SMO1*, which encodes a GTPase-activating protein, most similar to *GapA* in *Aspergillus nidulans*. We show that *SMO1* is necessary for the determination of conidial shape and the ability of spores to attach to hydrophobic substrates. Importantly, *SMO1* is also necessary for septin-mediated F-actin remodeling at the appressorium pore, and therefore plays a critical role in plant infection by the rice blast fungus.

## Materials and Methods

### Fungal strains, growth conditions, and DNA analysis

*M. oryzae* strains used in this study were the rice pathogens Guy11 (Leung *et al.* 1988) and a ∆*ku-70* mutant impaired in nonhomologous DNA-end joining (Kershaw and Talbot 2009), the weeping lovegrass pathogen 4091-5-8, and 12 *smo1* mutants (see Supplemental Material, Table S1), either spontaneous mutants selected as nonadherent, appressorium development, or spore shape mutants, or mutants generated by UV mutagenesis, from the original study (Hamer *et al.* 1989b). These strains had been stored as desiccated, frozen filter paper stocks for the 25 years preceding this study. Filter papers from these stocks were placed on complete medium agar (Talbot *et al.* 1993) and the fungus grown for 12 days before preparation of liquid cultures from which mycelium was recovered for DNA extraction. Growth, maintenance of *M. oryzae*, media composition, nucleic acid extraction, and transformation were all as described previously (Talbot *et al.* 1993). Gel electrophoresis, restriction enzyme digestion (routinely purchased from Promega, Madison, WI), gel blots, DNA manipulation, and sequencing were performed using standard procedures (Sambrook *et al.* 1989).

### Sequencing and SNP analysis

Genomic DNA was extracted from 12 *smo1* mutant strains and sequenced using a HiSeq 2500 (Illumina), generating 100 base paired-end reads. Reads were filtered using the fastq-mcf program from the ea-utils package (http://code.google.com/p/ea-utils/). Filtered reads were mapped against the *M. oryzae* (strain 70-15) reference genome version 8 (Dean *et al.* 2005), (https://fungi.ensembl.org/Magnaporthe_oryzae/Info/Annotation/) using the Burrows–Wheeler Aligner (Li and Durbin 2009) and coverage details are provided in Table S1. Bespoke Perl scripts were used to calculate mean aligned coverage of reads against the reference genome, to discover SNPs (based on minimum read depth of 10 and minimum base identity of 95%) and to identify genes in which SNPs occurred. Table S1 shows details of raw DNA sequence information generated from each mutant strain. The Integrative Genome Viewer (Robinson *et al.* 2011) was used to manually inspect read alignments for evidence of mutations in each strain. Sequence data for each mutant was submitted to the European Nucleotide Archive (ENA) database (http://www.ebi.ac.uk/ena/data/view/PRJEB27449), and accession numbers are listed in Table S1.

### Bulked segregant genome analysis

BSA (Michelmore *et al.* 1991) was performed on a segregating ascospore population. Genetic crosses were performed as described previously (Valent *et al.* 1991). Briefly, the two rice pathogenic strains 4395-4-4 (*smo1 alb1 Mat1-2*) and wild-type strain TH3 (*Mat1-1*) were inoculated together on oatmeal agar, grown aseptically at 24° for 7 days, and then at 20° until flask-shaped perithecia were visible at the mycelial junction. Perithecia were transferred to 4% distilled water agar, separated from all conidia, and broken open to reveal asci. Mature asci were removed with a glass needle and ascospores dissected from them. Ascospores were transferred individually to a 48-well plate containing complete medium and incubated for 4–5 days (Talbot *et al.* 1996). At this time, monoconidial reisolations were made from each well by picking individual conidia using a mounted glass needle and removing them to individual complete medium agar plates for growth at 24° for 12 days. Progeny were screened by microscopy and genomic DNA extracted from mycelial cultures of progeny using the CTAB method, described previously (Talbot *et al.* 1993). DNA samples were bulked into two samples; wild-type progeny and mutant progeny, and sequenced using HiSeq. Sequenced reads were aligned against the *M. oryzae* reference strain (70–15) assembly and examined for occurrence of SNPs segregating with the *smo1* mutation, as described above.

### Generation of targeted deletion mutants and strains expressing GFP fusions

Targeted gene replacement was carried out using a split-marker strategy (Catlett *et al.* 2003). Vectors were constructed using a hygromycin B resistance selectable marker, *hph* (Sweigard *et al.* 1997). To amplify split *hph* templates, the primers used were M13F with HY and M13R with YG, as described previously (Kershaw and Talbot 2009). Sequence data for the *SMO1* candidate gene, MGG_03846, was retrieved from the *M. oryzae* genome database (https://fungi.ensembl.org/Magnaporthe_oryzae/) and used to design specific primer pairs (5′-Smo50.1/3′-SmoM13f/and 5′-Smo30.1/3′-SmoM13r) to amplify regions flanking the open reading frame of MGG_03846 (Table S2). *M. oryzae* strain Guy-11 was transformed with the deletion cassettes (2 μg of DNA of each flank) and transformants selected in the presence of hygromycin B (200 μg ml^−1^). Two independent deletion mutants were obtained, as assessed by Southern blot analysis. A translational C-terminal MGG_03846 GFP fusion construct was generated by in-fusion cloning based on *in vitro* homologous recombination (Takara Clontech, San Germain-en-Laye, France). The primers 5′-Smop and 3′-SmoGFP were used to amplify a 4.5-kb fragment, which included 1.9 kb of the MGG_03846 promoter region and 2.6 kb of the MGG_03846 open reading frame minus the stop codon. A 1.4-kb GFP fragment with trpC terminator was amplified using primers 5′-smoGFP and 3′-TrpC, as listed in Table S2. Amplicons were cloned into pCB1532 (Sweigard *et al.* 1997), linearized with *Bam*HI and *Hin*dIII, which carries the *ILV1* cassette conferring resistance to sulfonylurea. Homologous recombination results in the assembly of fragments (5.9 kb) in the correct orientation to generate a gene fusion construct of 11.2 kb. The construct was transformed into the wild-type strain Guy11. For complementation of a Δ*smo1* mutant, the *SMO1-GFP* fusion cassette was transformed into the Δ*smo1-3* deletion mutant. A full-length 5.6-kb fragment of the *SMO1* gene from Guy11 was amplified with primers 5′-Smop and 3′-smo30.1 (Table S2), cloned into pCB1532 (Sweigard *et al.* 1997), and this construct was transformed into the *smo1* mutant CP751 (Hamer *et al.* 1989b). Transformants were selected in the presence of sulfonylurea (50 μg ml^−1^). For localization of fluorescent fusion proteins in the Δ*smo1* mutant, *SEP3-GFP* (Dagdas *et al.* 2012), *Gelsolin-GFP* (Ryder *et al.* 2013), *Lifeact-GFP* (Berepiki *et al.* 2010), *GFP-ATG8* (Kershaw and Talbot 2009), *^1^H-RFP* (tdTomato) (Saunders *et al.* 2010), and β*-tubulin:sGFP* (Saunders *et al.* 2010) constructs were transformed into Δ*smo1*, and transformants selected on either sulfonylurea (50 μg ml^−1^) or bialophos (50 μg ml^−1^).

### Appressorium development, penetration assays, and rice infections

*M. oryzae* conidia were obtained by harvesting suspensions in water from the surface of 12-day-old plate cultures prepared on complete medium (CM) agar. Infection-related development was assessed by incubating conidia on hydrophobic glass coverslips and allowing appressoria to form, before visualization by epifluorescence or laser confocal microscopy. To visualize STM, FITC-conjugated concanavalin (FITC-ConA) was added at 1μg ml^−1^ to harvested conidia and incubated at 24^ο^ for 20 min before examination. Rice leaf sheath (*Oryza sativa*) inoculations were performed, as described previously, using the susceptible rice cultivar CO-39 (Kankanala *et al.* 2007). Appressorium-mediated penetration of onion (*Allium cepa*) epidermal strips was assayed, as described previously (Balhadère *et al.* 1999), and assessed by recording the frequency of hyphal penetration from an appressorium. An incipient cytorrhysis assay was carried out by allowing appressoria to form in water on borosilicate cover slips for 24 hr, after which the water was replaced with a range of aqueous glycerol ranging from 0.25 to 2.5M and, after 30 min, the frequency of cytorrhysis determined (de Jong *et al.* 1997). Plant infection assays were performed by spraying seedlings of rice cultivar CO-39 with a suspension of 10^5^ conidia ml^−1^, as previously described (Talbot *et al.* 1993). Occurrence of blast symptoms was recorded 5 days after inoculation and experiments were performed three times.

### Protein–protein interaction studies

A yeast two-hybrid screen was performed to determine physical interactions of Smo1 and to investigate its function as a RasGAP, using the Matchmaker GAL4 Two-Hybrid system 3 (Takara Clontech). *SMO1*, *RAS2*, and *RAS1* cDNA were cloned into the bait vector pGBKT7 using primer combinations 5′-smoGB/3′-smoGB, 5′-ras2GB/3′ras2GB, and 5′-ras1GB/ras1GB. *SMO1*, *RAS2*, and *GEF1* cDNA were cloned into the prey vector PGADT7 using primer combinations 5′-smoGA/3′-smoGA, 5′-ras2GA/3′ras2GA, and 5′-gef1GA/gef1GA. Cloning was performed using in-fusion cloning (Takara Clontech). Sequencing was performed to ensure that constructs were in-frame (MWG operon). Yeast two-hybrid analysis was then carried out using the Matchmaker GAL4 Two-Hybrid system 3 (Takara Clontech) according to the manufacturer’s instructions (Wilson *et al.* 2010). For *in vivo* co-immunoprecipitation studies, total protein was extracted from lyophilized *M. oryzae* mycelium of strains expressing Smo1:GFP and ToxA:GFP (control) after growth in liquid CM for 48 hr. Protein extracts were co-immunoprecipitated using the GFP-Trap protocol, according to the manufacturer’s protocol (ChromoTek). Protein extracts were prepared for liquid chromatography-tandem mass spectrometry (LC-MS/MS) and separated by SDS-PAGE. Gels were cut into slices and LC-MS/MS analysis was performed at the University of Bristol Proteomics Facility.

### Light and epifluorescence microscopy

Epifluorescence microscopy was used to visualize the localization of fluorescent fusion proteins expressing enhanced GFP or red fluorescent protein (RFP) using an IX81 motorized inverted microscope (Olympus Microscopy UK, Southend-on-Sea, UK) equipped with a UPlanSApo 100X/1.40 Oil objective (Olympus). Excitation of fluorescently labeled proteins was carried out using a VS-LMS4 Laser-Merge-System with solid-state lasers (488 nm/50 mW). Laser intensity was controlled by a VS-AOTF100 System and coupled into the light path using a VS-20 Laser-Lens-System (Visitron Systems GmbH, Puchheim, Germany). Images were captured using a Charged-Coupled Device camera (Photometric CoolSNAP HQ2, Roper Scientific). All parts of the system were under the control of the software package MetaMorph (Molecular Devices, Downingtown, PA). High-resolution imaging of *M. oryzae* Tub2-GFP-expressing transformants was performed using a Leica SP8 laser confocal microscope, with an argon laser line (488 nm) to excite GFP for imaging.

### Data availability

The authors affirm that all data necessary for confirming the conclusions of the article are present within the article, figures, and tables. Strains and plasmids are available from the corresponding author upon request. Genome sequence data are available at the ENA; the accession numbers are listed in Table S1. Supplemental material available at Figshare: https://doi.org/10.25386/genetics.7312151.

## Results

### Identification of the SMO1 locus

**Figure 1:**
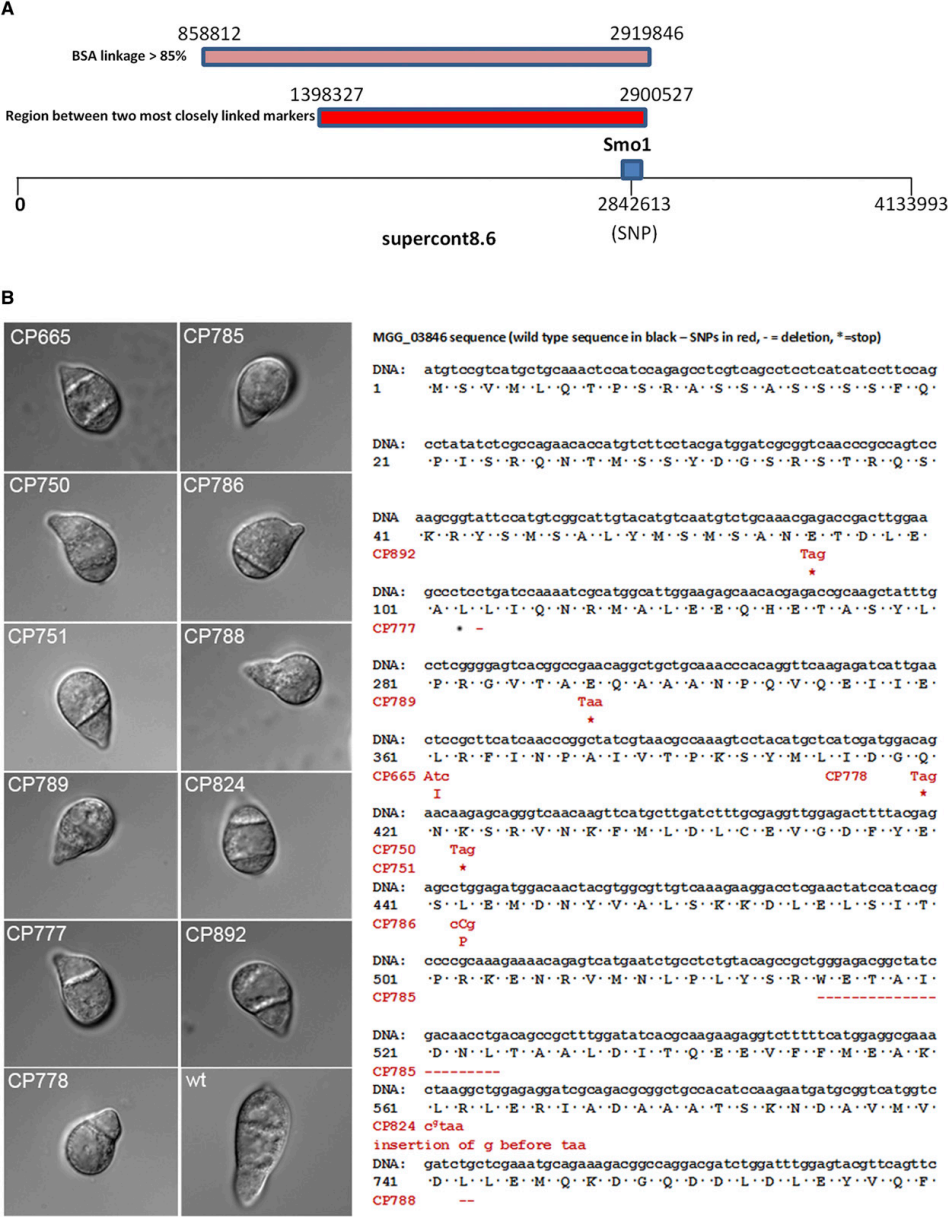
Identification of the *SMO1* locus in *M. oryzae*. (A) The identified *SMO1* gene lies on supercontig 8.6 (chromosome 6: 2,838,047–2,844,822, Ensembl database). Single-copy RFLP probes JH4.28 and JH5.00, which have previously been shown to be closely linked to the *SMO1* locus (Hamer and Givan 1990; Romao and Hamer 1992), were sequenced and mapped to the *M. oryzae* genome, and also found on supercontig 8.6, separated by a distance of 1,502,200 bases. A region in a similar position on supercontig 8.6 was defined by SNPs that showed a > 85% linkage to *SMO1* using bulk segregant analysis (BSA). (B) Micrographs showing spore morphology of *smo1* strains used in this study compared to wild-type strain Guy11 (bars, 10 μm). Smo1 mutants were originally obtained spontaneously or after UV mutagenesis (Hamer *et al.* 1989b). Nucleotide and amino acid sequences of MGG_03846 , indicating positions of SNPs and subsequent mutation in each of the *smo1* mutants. Wild-type sequence in black, SNPs in red, * = stop codon.

To identify the *SMO1* locus, we carried out BSA (Michelmore *et al.* 1991) using whole-genome sequencing to identify SNPs. *M. oryzae* rice pathogenic strain 4395-4-4 (*smo1 alb1 Mat1-2*) was crossed with a wild-type rice pathogenic strain TH3 (*Mat1-1*) and a total of 50 ascospores collected. Ascospore progeny were phenotypically characterized based on spore shape, as *smo1* mutants have one- or two-celled spherical or misshapen conidia, compared to the three-celled pyriform wild-type conidia. Progeny were therefore selected according to the Smo1 phenotype, DNA extracted from each individual, and then bulked. Whole-genome sequencing of bulked DNA samples identified a region of 2,061,034 bases on supercontig 8.6, which was defined by SNPs showing > 85% linkage to *SMO1* using BSA (Figure 1A). This conformed to the region originally defined by MGR586-based genetic mapping spanning the *SMO1* locus (Romao and Hamer 1992). Consistent with this, two single-copy RFLP probes previously shown to be closely linked to the *SMO1* locus (Hamer and Givan 1990; Romao and Hamer 1992), JH4.28 and JH5.00, were sequenced and mapped to the *M. oryzae* genome. They were both found on supercontig 8.6, separated by a distance of 1,502,200 bases. The identified *SMO1* gene lies within this region, as identified by BSA (Figure 1A).

We then analyzed an allelic series of original *smo1* mutants, which were either selected by UV mutagenesis as nonadherent mutants, appressorium development mutants, or spontaneous Smo1 mutants (Hamer *et al.* 1989b), as described in Table S1, and confirmed their phenotypes by spore morphology, as shown in Figure 1B. Genomic DNA was extracted from each strain and whole-genome sequencing carried out. We also carried out genome sequence analysis of the parental 4091-5-8 strain, from which the Smo1 mutants were originally selected (Hamer *et al.* 1989b). All Smo1 mutants shared 202,020 SNPs, which distinguished 4091-5-8 from the *M. oryzae* genome reference strain 70-15. Subtracting these shared SNPs from the derived SNP data sets for each strain identified SNPs unique to each mutant. Six of the 10 mutant *smo1* strains possessed a SNP in gene MGG_03846 . Manual inspection of reads aligned to MGG_03846 identified mutations in five other mutant strains (Figure 1B). Only CP790 did not contain a mutation in MGG_03846 , and our analysis suggests that this strain does not exhibit the Smo1 phenotype and therefore may have reverted since the original study (Hamer *et al.* 1989b). With the exception of strains CP750 and CP751, which had identical SNPs, the mutations were different in each strain, consistent with the allelic variability reported originally (Hamer *et al.* 1989b). Five of the strains had a SNP that introduces a stop codon into the open reading frame (nonsense mutation), four had either an insertion or deletion that produces a frameshift mutation, and two possessed a base pair substitution that resulted in a change in an amino acid residue (Figure 1B). The mutation in CP786, for instance, changes a leucine to proline, which is likely to produce a distinct alteration in secondary structure, given that proline acts as a structural disruptor in the middle of regular secondary structure elements such as α-helices and β-sheets (Williamson 1994).

### Cloning and characterization of the SMO1 gene of *M. oryzae*

The *SMO1* candidate gene MGG_03846 is 2643 bp in length with four introns of 81, 83, 78, and 65 bp, respectively, and encodes a putative 780-aa protein. Bioinformatic analysis predicted that MGG_03846 encodes a Ras GTPase-activating protein (RasGAP), and the predicted gene product possesses two domains: a GTPase activator domain for Ras-like GTPase from amino acids 193–401 and a RASGAP C-terminal motif from amino acids 580–699. There are four putative GapA-encoding genes in *M. oryzae*, and phylogenetic analysis (Figure S1) revealed potential *SMO1* orthologs (Larkin et al. 2007) of *GapA* from *A. nidulans* (Harispe *et al.* 2008) and *Gap1* from *Schizosaccharomyces pombe* (Imai *et al.* 1991). Phylogenetic analysis of other putative GAPs in *M. oryzae* suggests that MGG_03700 is a homolog of the *Saccharomyces cerevisiae* IQG1, which controls actin-ring formation and cytokinesis (Epp and Chant 1997). MGG_08105 is a homolog of the *S. cerevisiae BUD2*, which plays a role in spindle position checkpoint and bud site selection (Park *et al.* 1993), while MGG_11425 is a homolog of the *S. cerevisiae* RasGAPs *IRA1* and *IRA2*, which are negative regulators of the Ras-cAMP signaling pathway required for reducing cAMP levels under nutrient-limiting conditions (Tanaka *et al.* 1989, 1990) (Figure S1).

To determine whether the candidate RASGAP-encoding gene is *SMO1*, we cloned a full-length copy of MGG_03846 under control of its native promoter and transformed this into *smo1* mutant CP750 (Hamer *et al.* 1989b). Reintroduction of *SMO1* fully complemented the *smo1* spore shape phenotype, and spores in the complemented strain germinated to produce short germ tubes and normal appressoria (Figure S2).

### Targeted deletion of SMO1 leads to cell shape defects

**Figure 2:**
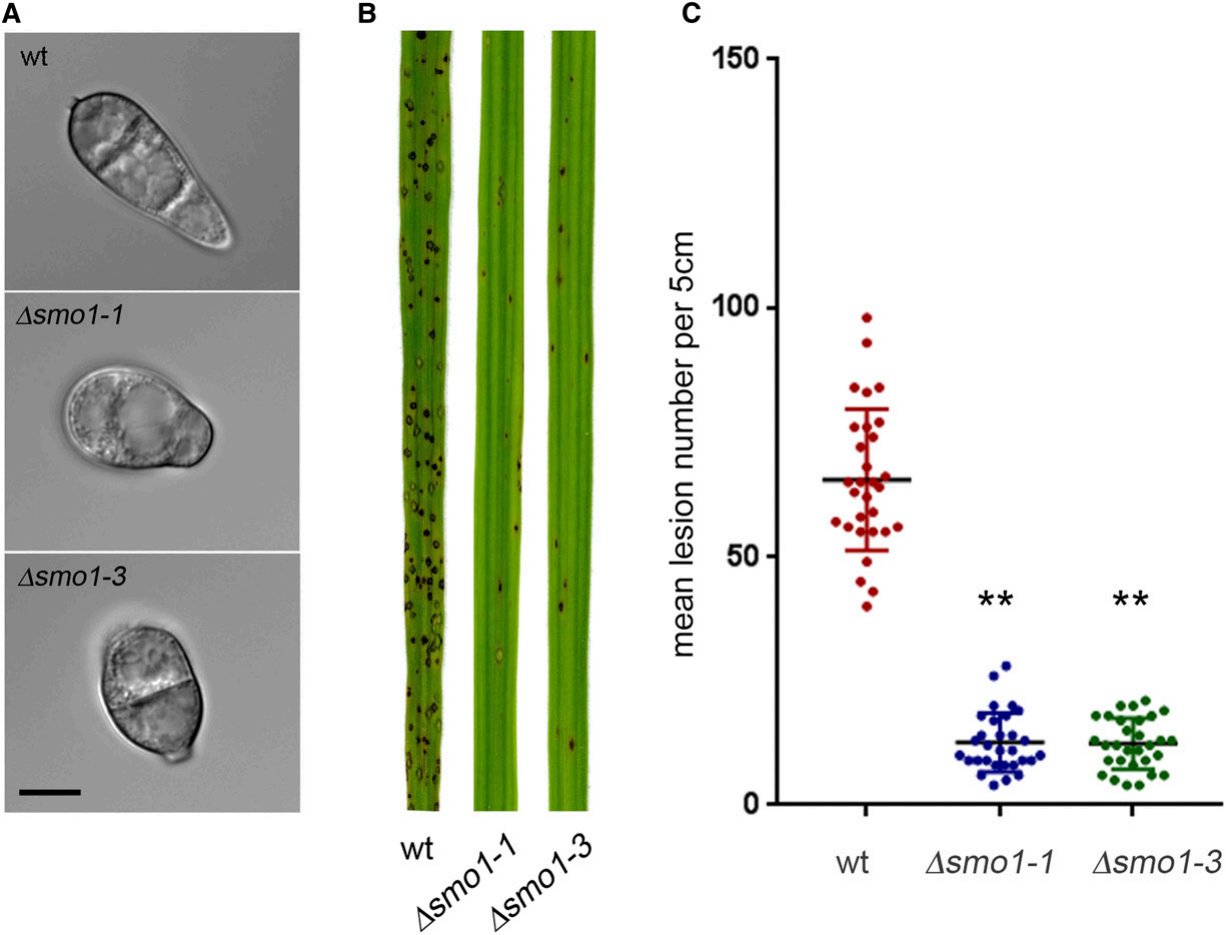
Deletion of MGG_03846 resulted in *smo1* spore phenotype and mutants were reduced in plant infection. (A) Micrographs showing spores of two MGG_03846 deletion strains (Δ*smo1*) as compared to wild-type (wt) strain Guy11 (bar, 5 μm). (B) Photographs of leaves from infected plants. Seedlings of rice cultivar CO-39 were inoculated with conidial suspensions (5 × 10^4^ ml^−1^). Seedlings were incubated for 5 days to allow symptom development. (C) Box plot of mean lesion density per 5-cm leaf after infection with MGG_03846 deletion mutants compared to wild-type. Error bar equals SE of the mean. * *P* < 0.0001 [unpaired Students’s *t*-test (*n* = 3 experiments of 40 leaves)].

**Figure 3:**
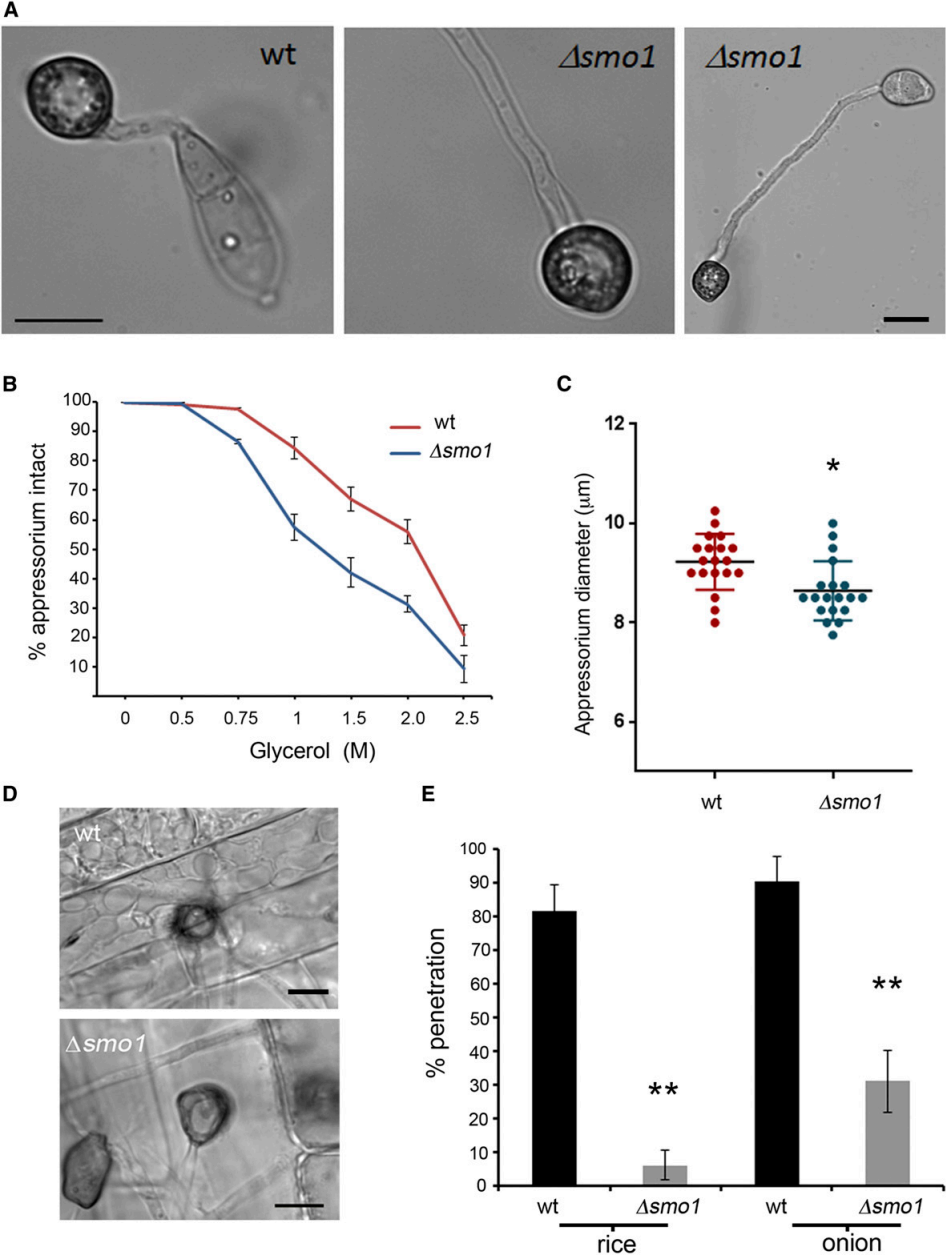
The Δ*smo1* mutant is able to elaborate an appressorium, which is impaired in function. (A) Micrograph showing Δ*smo1* mutant with extended germ tube compared to wild-type (wt) strain (bar, 5 μm). (B) Incipient cytorrhysis assays measuring intracellular glycerol were carried out by allowing appressoria to form in water on borosilicate cover slips for 24 hr, after which the water was replaced with a range of aqueous glycerol solutions ranging from 0.25 to 2.5 M. The rate of cell collapse was determined after 30 min. A concentration of 1.25 M glycerol caused the collapse of 50% of appressoria in the Δ*smo1* mutant whereas 2.25 M glycerol was required for 50% collapse in the wild-type. *P* < 0.0001 (unpaired Student’s *t*-test, *n* = 100). (C) Box plot showing reduced appressorium diameter of Δ*smo1* mutant compared to wild-type, Guy11. *P < 0.01 (unpaired Student’s *t*-test, *n* = 50). (D) Micrograph comparing penetration of rice leaf sheath by Guy11 and the Δ*smo1* mutant. Inoculations were performed as described previously using the susceptible rice line CO-39. (E) Bar chart showing percentage penetration of Guy11 and Δ*smo1* mutant on leaf sheath and onion epidermis, assessed by recording the frequency of hyphal penetration from an appressorium ** *P* < 0.001 (unpaired Student’s *t*-test, *n* = 24).

We next carried out targeted deletion to generate a Δ*smo1* mutant in the wild-type, rice pathogenic strain of *M. oryzae*, Guy11, and two independent deletion strains were selected and confirmed by Southern blot analysis for further analysis. The morphology of Δ*smo1* mutants was similar to the original *smo1* mutants, with mycelial colonies more compact, white, and fluffy compared to those of the isogenic wild-type strain Guy-11. Conidia were more rounded and predominantly unicellular, or two-celled (Figure 2A). By contrast, wild-type spores are typically 22–25 μm in length, and 8.75 and 5.5 μm in diameter, whereas Δ*smo1* conidia are typically 12.5–15 μm in length, and 8.5–10 μm in diameter. Conidia of Δ*smo1* mutants germinate normally, but produce longer germ tubes than the wild-type, typically 87 μm in Δ*smo1* compared to 22 μm in Guy11 (Figure 3A). Appressorium development was delayed, and appressoria were typically misshapen and slightly smaller, 8.64 ± 0.56 μm in diameter in Δ*smo1*, compared to 9.55 ± 0.56 μm in Guy11 (*P* < 0.01), as shown in Figure 3.

As a consequence of the abnormal spore morphology and delay in appressorium formation in Δ*smo1* mutants, we decided to investigate the pattern of nuclear division during appressorium development. In *M. oryzae*, a single round of mitosis occurs prior to appressorium development, followed by conidial cell death and the degradation of nuclei in each conidial cell (Veneault-Fourrey *et al.* 2006; Saunders *et al.* 2010). We introduced an ^1^H-RFP fusion into the Δ*smo1* mutant to visualize nuclear dynamics by live-cell imaging (Figure S3A). Nuclear division in Δ*smo1* takes place within 4–6 hr postinoculation (hpi), as observed in Guy11, and, in addition, one daughter nucleus migrates to the developing appressorium and the other nucleus returned to the conidium, in the same way as the wild-type (Figure S3A). However, nuclear material was often observed in the longer germ tube as well as the conidium. We used Calcofluor-white to examine septation events in the germ tube and observed that one septum normally forms in the germ tube, often near to the conidium (Figure S3B). After 16 hr, nuclei in Δ*smo1* conidia start to degrade and by 24 hr the spore had collapsed, as was observed in Guy11 (Figure S3A). We conclude that Δ*smo1* mutants show defects in spore shape and organization, and exhibit extended germ tube growth associated with a delay in appressorium development.

### The SMO1 gene encodes a virulence factor in *M. oryzae*

To determine the role of *SMO1* in fungal pathogenicity, we inoculated the susceptible rice cultivar CO-39 with spore suspensions of two Δ*smo1* mutant strains and Guy11. The Δ*smo1* mutants generated significantly reduced numbers of disease lesions, 13.44 ± 1.34 per 5-cm leaf, compared to 65 ± 6.94 lesions per 5-cm leaf in Guy11 (*P* < 0.0001) (Figure 2, B and C). To determine whether the reduced ability of Δ*smo1* mutants to cause disease lesions was due to reduced appressorium turgor, we incubated appressoria of Δ*smo1* mutants in a series of glycerol solutions of increasing molarity and measured the frequency of incipient cytorrhysis (cell collapse) (Howard *et al.* 1991). A concentration of 1.25 M glycerol caused the collapse of 50% of appressoria in the Δ*smo1* mutant whereas in the wild-type 2.25 M glycerol was required for 50% (*P* < 0.0001) (Figure 3C). This reduction in turgor is consistent with an appressorium penetration defect. We therefore applied the Δ*smo1* mutant to excised rice leaf sheath to determine the frequency of appressorium-mediated penetration. We observed that Guy11 had a frequency of successful penetration events of 81.66% ± 7.59 penetration, compared to 6.3 ± 4.37 in Δ*smo1* (*P* < 0.001), as shown in Figure 3, D and E. We also tested whether Δ*smo1* mutants were able to penetrate onion epidermis and found slightly increased penetration compared to that of Δ*smo1* mutants on rice leaf sheaths, but this was still significantly reduced compared to wild-type (90.33. ± 7.59 compared to 31 ± 9.23 in Δ*smo1*; *P* < 0.001), as shown in Figure 3E. We conclude that Δ*smo1* mutants are reduced in their ability to cause rice blast disease because of impairment in appressorium function, including a reduction in turgor and the frequency of penetration peg development.

### Smo1 localizes to the appressorium pore during plant infection

**Figure 4:**
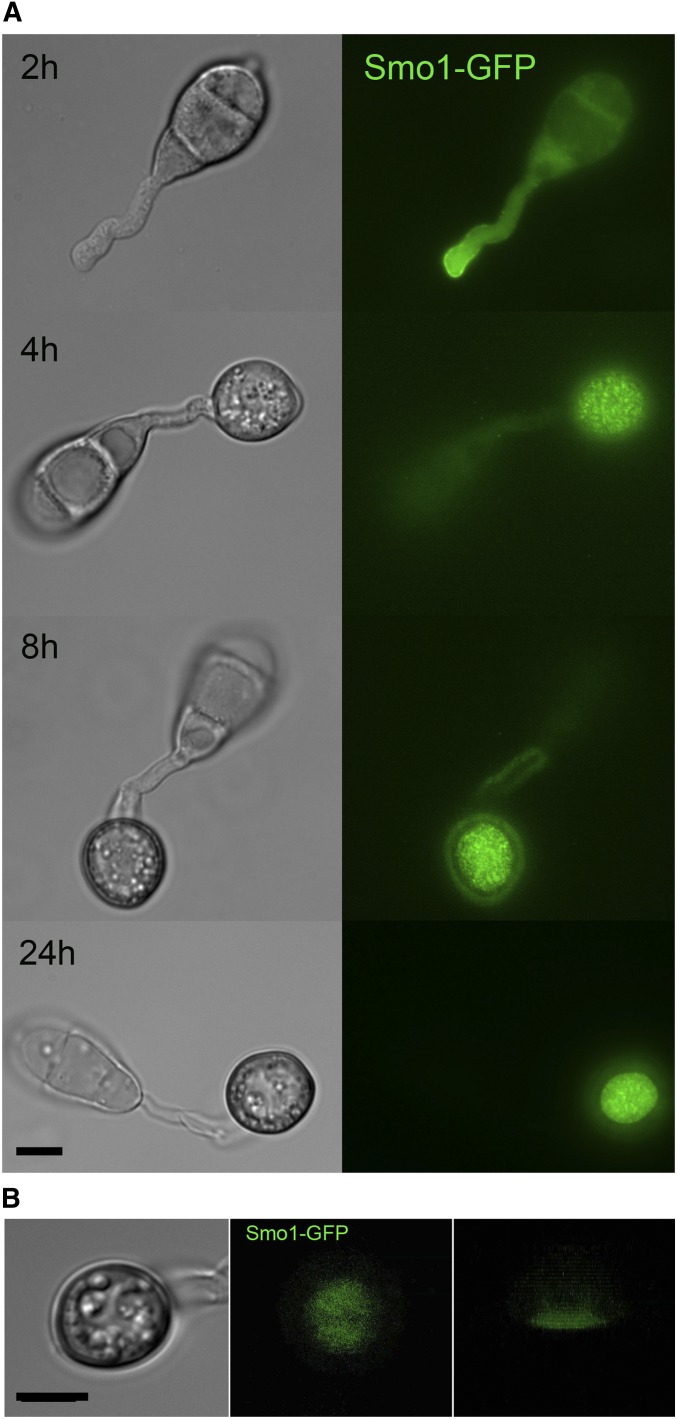
Live-cell imaging of *M. oryzae* wild-type strain expressing Smo1-GFP (A) Cellular localization of Smo1-GFP at the tip of the germ tube and as punctate structures in both the developing appressoria. (B) Distribution of Smo1-GFP in mature appressorium visualized by laser confocal microscopy. The right-hand panel shows confocal image of transverse view of appressorium with Smo1-GFP present predominantly at the appressorium–substrate interface (see also Movie S1). Spores were harvested and inoculated onto hydrophobic cover slips, and visualized by epifluorescence or laser confocal microscopy. Bar, 10 μm.

To determine the subcellular localization of the Smo1 protein and its temporal dynamics during infection-related development, we generated a *SMO1-GFP* fusion that was introduced into Guy11 and a Δ*smo1* mutant. Expression of *SMO1-GFP* in the Δ*smo1* mutant strain was sufficient to restore wild-type spore and appressorium morphologies, and the ability to infect rice and cause disease (Figure S2). Analysis of the cellular localization showed that Smo1 localizes to the tip of germ tubes during germination. As the appressorium forms, Smo1 localized initially as small puncta throughout the appressorium (Figure 4A). However, after 24 hr when maximal turgor is established in the appressorium, Smo1 localization became more condensed and by using three-dimensional reconstruction of a mature appressorium, Smo1-GFP was observed to localize predominantly to the base of the appressorium around the appressorium pore (Figure 4B and Movie S1). Smo1 distribution is therefore associated with regions of polarized growth, such as the germ tube tip. In the appressorium, Smo1 localizes to the point at which anisotropic growth is reestablished for penetration peg development and plant infection.

### Δ*smo1* mutants are defective in STM generation and surface attachment

**Figure 5:**
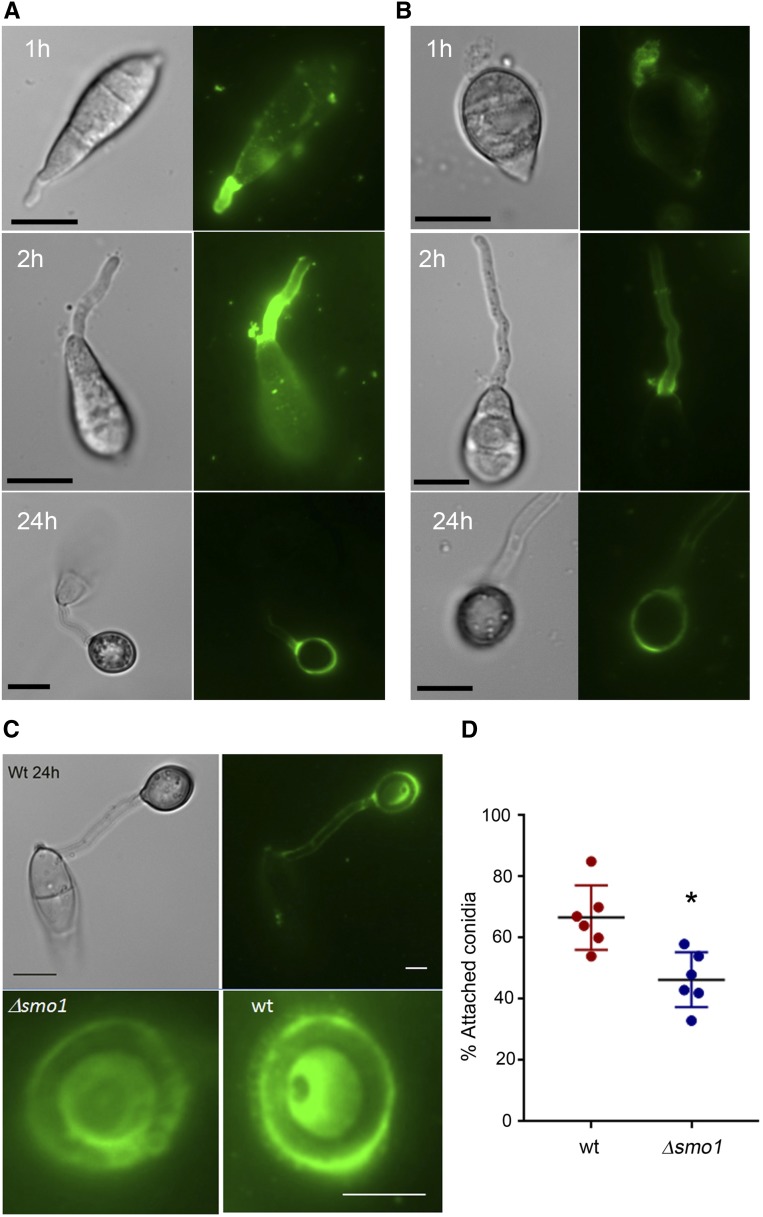
Live-cell imaging of *M. oryza*e wild-type (wt) and Δ*smo1* mutant, showing release and levels of spore tip mucilage by addition of FITC-concanavalin to germinating *M. oryzae* conidia of (A) Guy11 and (B) Δ*smo1* mutants. Spores were harvested and inoculated onto hydrophobic cover slips before addition of FITC-concanavalin (100 μg ml^−1^). Bar, 10 μm. (C) FITC-concanavalin staining of mucilage in mature appressoria of Guy11 and Δ*smo1* mutants after 24 hr. Bar, 5 μm. (D) Box plot showing percentage of germinated conidia of Guy11 and Δ*smo1* remaining attached to cover slips after washing. Conidia were harvested and counted (1 × 10^5^ ml^−1^), and allowed to attach to hydrophobic cover slips 30 min before washing with water. * *P* < 0.01 (unpaired Student’s *t*-test, *n* = 100).

The tight adhesion of conidia to the rice leaf surface is critical for rice blast disease and involves the release of STM from the tip of the conidium (Hamer *et al.* 1988) prior to germination. We used FITC-ConA to compare STM released from spores of Guy-11 and the Δ*smo1* mutant. We evaluated levels of STM secretion between 1 and 24 hr postinoculation. The adhesive is released from the conidial apex initially and then the germ tube tip (Hamer *et al.* 1988). This revealed a clear reduction in STM secretion in Δ*smo1* mutants compared to Guy-11. During the early stages of conidial attachment and germination, STM in the Δ*smo1* mutant was noticeably reduced compared to Guy-11 (Figure 5, A and B). We also observed Guy-11 and Δ*smo1* mutants after 24 hr and observed a similar reduction in a ConA-positive mucilage layer around the mature appressorium in a Δ*smo1* mutant (Figure 5C). To examine conidial adhesion, we then counted the number of conidia that could be removed from the surface of hydrophobic coverslips by washing 30 min after inoculation. In Guy11, 67 ± 6.9% of conidia remained attached to polytetrafluoroethylene (PTFE) Teflon surfaces after washing, whereas in Δ*smo1* mutants, 48.4 ± 9.97% (*P* < 0.01) remained attached (Figure 5D). We conclude that STM secretion is impaired in Δ*smo1* mutants, which show reduced adhesion to hydrophobic surfaces.

### Δ*smo1* mutants are impaired in septin-mediated F-actin reorganization at the appressorium pore

**Figure 6:**
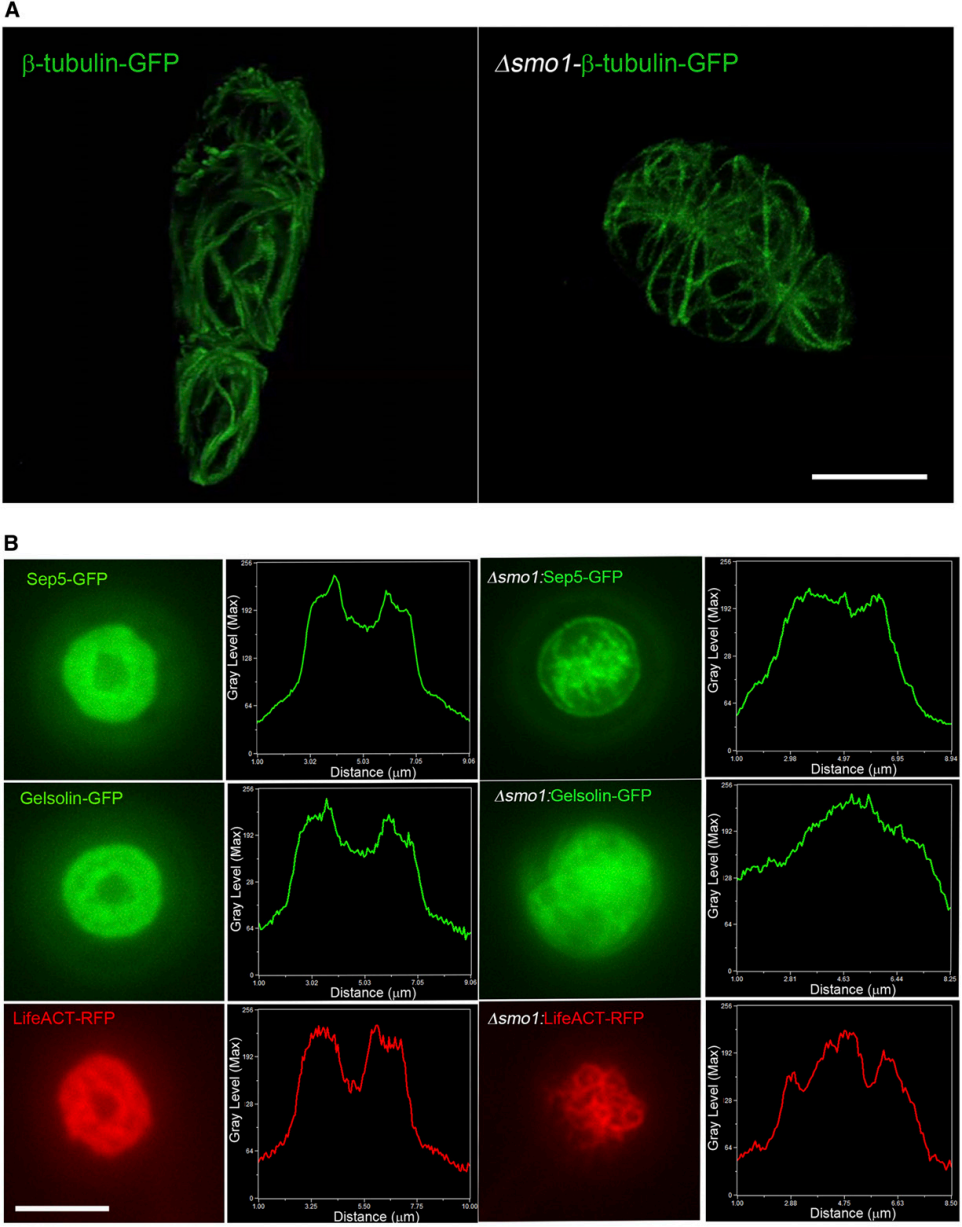
Δ*smo1* mutants are unable to undergo septin-mediated F-actin remodeling in the appressorium. (A) Expression of β-tubulin-GFP in conidia of *M. oryzae* Guy11 (left-hand panel) and Δ*smo1* mutant (right-hand panel). Microtubules showed aberrant distribution and organization, consistent with spore shape defect in Δ*smo1* mutant. (B) Live-cell imaging of septin-dependent F-actin network in appressoria of *M. oryzae* Guy11 and Δ*smo1* mutants at 24 hr postinoculation, visualized by laser-assisted epifluorescence microscopy. Localization of Sep5-GFP, Gelsolin-GFP, and LifeAct-red fluorescent protein (RFP) at the appressorium pore with corresponding line-scan graphs to show distribution of fluorescence signal in a transverse section. Organization of appressorium pore components requires Smo1. Bar, 5 μm.

The conidial shape phenotype of Δ*smo1* mutants suggested an effect on the distribution and organization of cytoskeletal components. We therefore visualized the distribution of microtubules based on expression of the β-tubulin Tub2-GFP fusion protein. Conidia of the wild-type Guy11 showed a network of long microtubules defining each of the three cells within the spore (Figure 6A and Movie S2). By contrast, microtubules observed in spores of a Δ*smo1* mutant showed an abnormal distribution, consistent with the spherical shape of spores (Figure 6A and Movie S3). A key requirement for appressorium function in *M. oryzae* is the recruitment and organization of a septin-dependent toroidal F-actin network at the appressorium pore. Septins provide cortical rigidity to the infection cell at the point of penetration and act as a diffusion barrier for the organization of polarity determinants required for penetration hypha development (Dagdas *et al.* 2012). We decided to investigate organization of the septin Sep5-GFP (Dagdas *et al.* 2012) in the Δ*smo1* mutant. In Guy11, a septin ring was visible surrounding the appressorium pore, but this was mislocalized in the Δ*smo1* mutant (Figure 6B). We therefore observed the F-actin cytoskeleton, by expressing actin-binding protein–gene fusions LifeAct-RFP and Gelsolin-GFP (Berepiki *et al.* 2010; Ryder *et al.* 2013) in the Δ*smo1* mutants and observing appressoria at 24 hpi. In Guy-11, Lifeact-GFP and Gelsolin-GFP fluorescence revealed the toroidal F-actin network at the appressorium pore, which marks the point at which the penetration peg emerges (Figure 6B). By contrast, the Δ*smo1* mutant showed dispersed and nonspecific localization of LifeAct-RFP and Gelsolin-GFP (Figure 6B). We conclude that the septin-mediated F-actin dynamics necessary for host penetration are regulated by signaling pathways acting downstream of Smo1.

### Protein–protein interaction studies to identify Smo1-interacting partners

**Figure 7:**
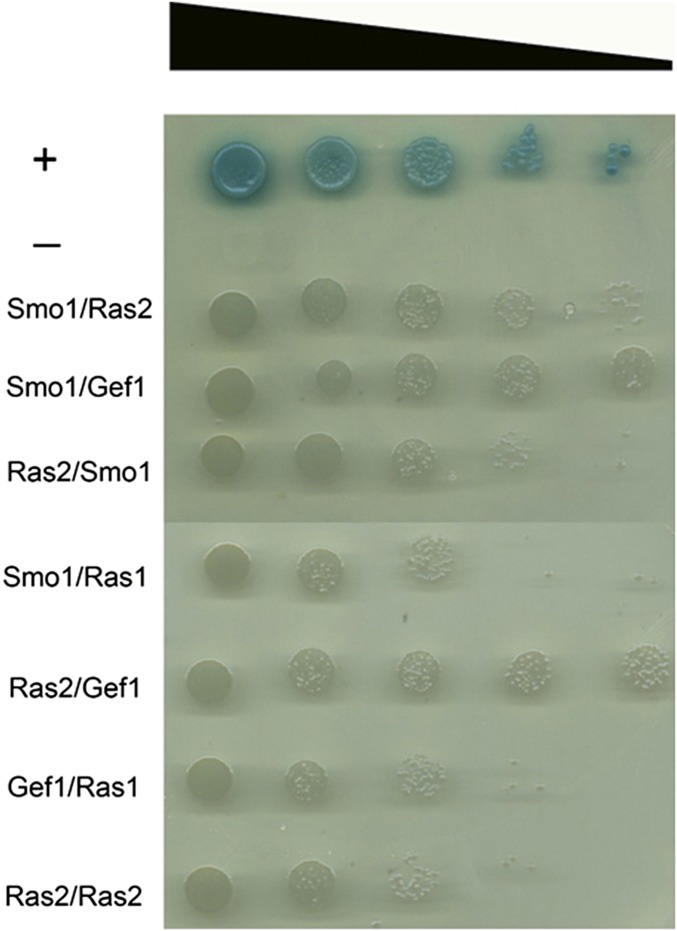
Yeast two-hybrid analysis. Yeast two-hybrid screens were performed to determine putative physical interactions of Smo1 and to confirm its function as a Ras-GAP. *SMO1*, *RAS2*, and *RAS1* cDNAs were cloned into the bait vector pGBKT7. *SMO1*, *RAS2*, and *GEF1* cDNA were cloned into the prey vector PGADT7. Simultaneous cotransformation of the pGBK-Ras2 (bait–Ras2) and pGAD-Smo1 (prey–Smo1) vectors into the yeast two-hybrid Gold strain results in the activation of all four reporter genes and growth on high-stringency media (-His/-Ade/-Leu/-Trp/+X-α-Gal). Smo1/Gef1 showed the highest-stringency interaction. +, positive control; −, negative empty prey vector control.

The identification of Smo1 as a putative RasGAP protein prompted us to identify its potential interacting partners. Two independent lines of investigation were followed. First of all, yeast two-hybrid analysis was carried out between Smo1 and confirmed Ras signaling components from *M. oryzae*. Initially, control experiments were performed in which pGAD-Smo1 (prey–Smo1), pGAD-Ras2 (prey–Ras2), pGAD-Gef1(prey–Smo1), pGBK-Ras(bait–Ras2), pGBK-Smo 1 (bait–Smo1), and pGBK-Ras1 (bait–Ras1) were independently transformed into the yeast two-hybrid Gold strain before plating onto SD/-Leu and SD/-Trp media, respectively. The lack of growth on these media demonstrates that none of the vectors are capable of auto-activating reporter genes. Simultaneous cotransformation of the pGBK-Ras2 (bait–Ras2) and pGAD-Smo1 (prey–Smo1) vectors into the Y2H Gold strain resulted in activation of all four reporter genes and growth on high-stringency medium (-His/-Ade/-Leu/-Trp/+X-α-Gal) (Figure 7). Cotransformation also activated *MEL1* expression, in which the enzyme α–galactosidase is secreted into the medium, resulting in hydrolysis of X-α-Gal in the medium and turning the yeast colony blue. Growth on such high-stringency media supports the hypothesis that Smo1 and Ras2 can physically interact. Putative interactions were also observed between Smo1 and Gef1, and between Ras2 and Gef1. Weaker interactions were also observed between Ras1 and both Gef1 and Smo1. When considered together, the interactions are consistent with Smo1 acting as a GTPase-activating protein on Ras2. We cannot preclude that Smo1 also plays a regulatory role in Ras1 signaling, but it shows much higher affinity to Ras2.

Second, we carried out co-immunoprecipitation of Smo1-GFP from hyphae of *M. oryzae* and identified interacting proteins by mass spectrometry. This revealed putative interactions with MAP kinase signaling pathway components, previously implicated in appressorium development, such as Mst11, Mst7, and Mst50, which all operate upstream of the Pmk1 MAP kinase, as well as the WD40 repeat protein Mip11, as shown in Table 1. Moreover, Smo1 appears to interact with the four core septins and with components of the exocyst complex, which are known to be associated with appressorium pore function (Dagdas *et al.* 2012; Gupta *et al.* 2015) as well as autophagy components that are also necessary for appressorium function (Table 1) (Kershaw and Talbot 2009).

**Table 1:** Putative Smo1-interacting proteins in *M. oryzae* identified by co-immunoprecipitation

		Total spectral count/total protein coverage (%)		
*M. oryzae* proteins identified by co-immunoprecipitation			Smo1:GFP*^a^*	Control
MAPK	Mst11	MGG_04100	2/2	1/1
	Mst7	MGG_06482	6/11	4/5
	Mst50	MGG_05199	2/2	1/1
	Hog1	MGG_01822	8/13	1/2
WD40 repeat	Mip11	MGG_04719	18/93	13/24
Mck1MEK kinase	Mip4	MGG_00883	2/2	0/0
MKK2 MEK	Mip5	MGG_06482	6/11	2/2
Histidine kinase	Mip7	MGG_11174	8/10	0/0
Ser/Thr phosphatase	Mip12	MGG_03838	6/14	0/0
G2/M control protein Sum2	Mip13	MGG_02405	1/1	0/0
				
Septins	Sep3	MGG_01521	11/21	4/5
	Sep4	MGG_06726	11/27	4/5
	Sep5	MGG_03087	12/18	6/7
	Sep6	MGG_07466	5/13	9/16
	Sep7	MGG_03087	12/18	6/7
				
Autophagy	Atg3	MGG_02959	1/1	0/0
	Atg4	MGG_03580	1/1	0/0
	Atg5	MGG_09262	1/1	0/0
	Atg7	MGG_07297	5/6	0/0
				
Exocyst	Sec24	MGG_09564	10/11	0/0
	Sec1	MGG_12345	4/4	0/0
	Sec8	MGG_03985	2/2	0/0
	Sec7	MGG_14173	12/20	2/2
	Sec31	MGG_06910	14/25	4/5
				
GAPs/GEFs	Smo1	MGG_03846	17/30	0/0
	Rho Gap	MGG_04377	1/1	0/0
	Rho GEF	MGG_12644	4/4	0/0
	RanGAP	MGG_01248	6/9	0/0
	Arf Gap	MGG_01472	3/4	0/0
				
Other	NoxR	MGG_05280	1/2	0/0
	Stu1	MGG_00692	1/1	0/0
Neutral trehalose		MGG_09471	4/5	0/0
Trehalose phosphatase		MGG_03441	27/56	4/7
Actin binding		MGG_03879	14/33	4/5
Myosin		MGG_00748	11/16	3/6
Scytalone dehydratase		MGG_05059	10/18	4/7
ATPase		MGG_04994	30/76	6/7
Aconitase hydratase		MGG_03521	30/134	8/12
Malate synthase		MGG_02813	22/60	0/0
Glutamate synthase		MGG_07187	60/108	00
	Arf6	MGG_10676	2/2	0/0

MEK, MAPK/ERK kinase; GAP, GTPase-activating protein; GEF, guanine nucleotide exchange factor.

a Co-immunoprecipitation of protein extracts from mycelium expressing Smo1-GFP or ToxA-GFP (control) using anti-GFP antibodies.

## Discussion

**Figure 8:**
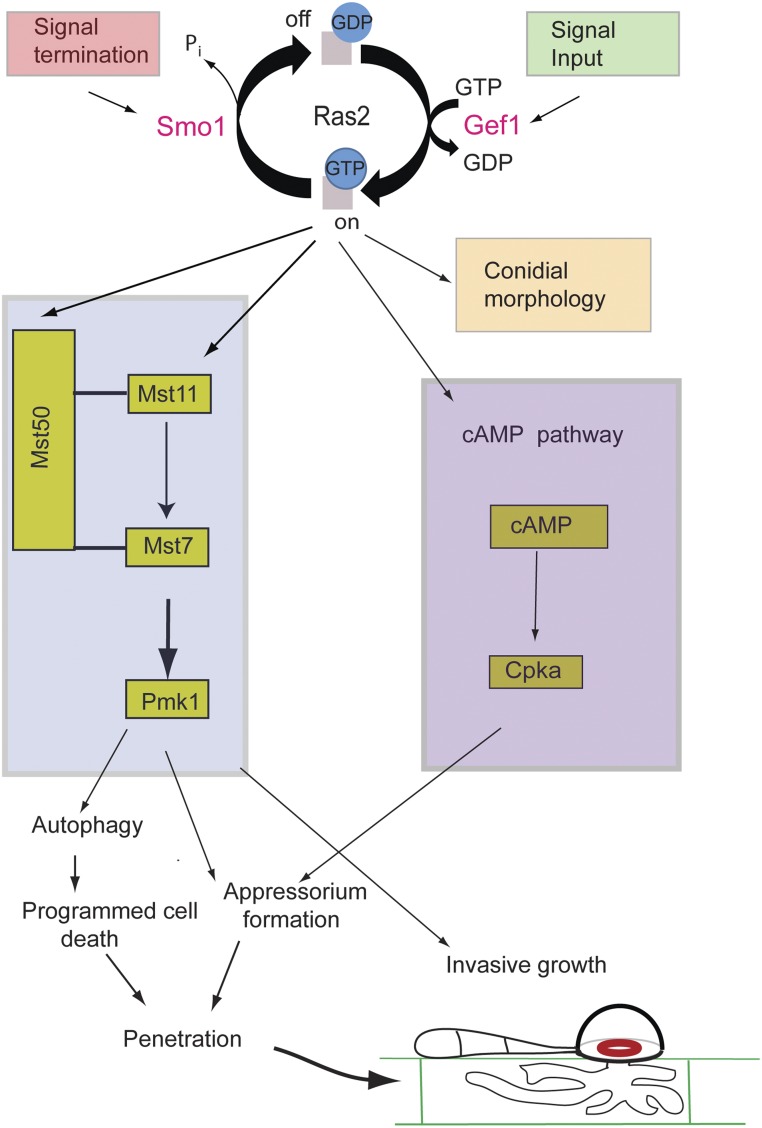
Model for the potential action of Smo1 in Ras2 signaling, and its regulation of septin-dependent appressorium repolarization and plant infection.

In this report, we have provided evidence that *SMO1* encodes a Ras GTPase-activating protein that plays important functions in cell shape determination and infection-related development in the rice blast fungus. *SMO1* is critical for rice blast disease, and plays a significant role in conidium and appressorium shape determination, and attachment to the leaf surface, in addition to an important regulatory function in the repolarization apparatus that operates within the appressorium. Smo1 is essential for septin recruitment and organization at the appressorium pore, which in turn is necessary for F-actin reorganization and penetration peg development (Dagdas *et al.* 2012). Smo1 physically interacts with Ras2, suggesting a model in which Ras signaling is required for appressorium repolarization, as shown in Figure 8.

Smo1 mutants were so frequently identified during the early days of rice blast molecular genetic analysis that Hamer and colleagues (1989b) suggested that the *SMO* locus might be highly mutable. For example, isolation of spontaneous mutants with melanin pigment defects and benomyl resistance identified double mutants that were also Smo1 (CP665 and CP892). Separate genetic screens to identify mutants with a defect in appressorium development or a search for STM mutants with reduced capacity to attach to hydrophobic surfaces also mainly identified Smo1 mutants (Hamer *et al.* 1989b). However, in spite of the rapid genetic mapping of the *SMO1* locus (Hamer and Givan 1990), the gene proved to be extremely difficult to clone and, after ∼6 years of effort, *SMO1* cloning was finally abandoned. Recent advances in genome sequencing, the availability of numerous independent Smo1^-^ mutants, and the ability to carry out genetic crosses readily in *M. oryzae*, have now enabled us to identify *SMO1* and to understand the genetic events leading to frequent loss of gene function. Surprisingly, mutational events leading to inactivation of *SMO1* were all SNPs or small insertions/deletions in the coding sequence. This result contrasts with frequent deletion of the highly mutable *BUF1* melanin biosynthesis gene, which presumably occurs by transposon-mediated recombination (Chumley and Valent 1991; Farman 2002). The *SMO1* gene does not reside in a particularly transposon-rich region of the genome. Therefore, mechanisms for frequent isolation of Smo mutants and the original difficulties in cloning *SMO1* remain to be explained. It is possible that the number of transposable elements and repeated DNA sequences normally distributed across the *M. oryzae* genome was sufficient to prevent map-based cloning efforts from being effective in the early 1990s, given the absence of extensive DNA sequence information, and large-insert genomic libraries (such as Bacterial Artificial Chromosome Libraries) at that time.

*SMO1* encodes a GTPase activating protein (GapA) involved in the regulation of Ras proteins, and is one of four found in the *M. oryzae* genome. RasGAPs work in conjunction with Ras-GEFs to regulate the activity of Ras proteins in response to external stimuli, affecting downstream signaling pathways necessary for the regulation of morphological transitions necessary for growth in eukaryotes (Boguski and McCormick 1993). For example, a RasGAP protein MadC, from the fungus *Phycomyces blakesleeanus*, has been shown to form part of the photosensory pathway for light-dependent fruiting body formation (Polaino *et al.* 2017).

We have shown by yeast two-hybrid analysis that Smo1 interacts with both Ras2 and Gef1, providing further evidence that Smo1 functions as a RasGAP and that it can form a complex with the corresponding guanine nucleotide exchange factor. Gap proteins are classified based on sequence homology within their Gap domains, with each domain specific for a class of G-proteins (Donovan *et al.* 2002). The different arrangements of domains, and in many cases the inclusion of distinct additional domains in the RasGAP family, suggest that these proteins are subject to a diverse range of cellular interactions (Donovan *et al.* 2002). In *M. oryzae*, four putative RasGAPs can be classified into four different clusters according to their sequences and domain structures. Smo1 possess two domains, a GTPase activation domain for Ras-like GTPase and a RASGAP C-terminal motif. These domains are characteristic of proteins belonging to the Ras-specific GAPs. The sequence placed Smo1 in a cluster with the *A. nidulans GapA* (Harispe *et al.* 2008) and *Gap1* from *Sc. pombe* (Imai *et al.* 1991). In *A. nidulans*, *GapA* mutants exhibit abnormal conidiophores, delayed polarity maintenance characterized by apical swelling, and subapical hyphal branching (Harispe *et al.* 2008). In addition, F-actin distribution is lost in Δ*gapA* cells, suggesting a role for *GapA* in the F-actin cytoskeleton organization required for hyphal growth (Harispe *et al.* 2008). Mutation of Gap1 in *Sc. pombe* results in hypersensitivity to the mating factor pheromone and the inability to perform efficient mating, which are identical phenotypes to those caused by activated ras1 mutations (Imai *et al.* 1991). The defect in polar growth suggests that *GAP1* is involved in polarity maintenance, working antagonistically with Ste6 in the regulation of Ras-GTPase in *Sc. pombe*. Involvement of a RasGAP in fungal morphogenesis was first reported for the basidiomycete white rot fungus *Schizophyllum commune*, in which *Gap1* deletion was shown to affect sexual development, with mutants unable to form gills on fruiting bodies and producing no basidiospores. In addition, growth phenotypes suggested involvement in the maintenance of polarity (Schubert *et al.* 2006). The Smo1 mutant phenotypes observed are therefore consistent with those of GAP genes identified in other fungi, with effects on cell shape determination, polar/nonpolar growth transitions, and regulation of the F-actin cytoskeleton.

Three other putative RasGAPs predicted in *M. oryzae*, MGG_11425.6, MGG_08105.6, and MGG_03700.6, have not yet been characterized. MGG_03700 is a homolog of the *S. cerevisiae* Iqg1, an essential gene shown by depletion and overexpression analysis to be required for cytokinesis and actin-ring formation (Epp and Chant 1997). Iqg1 possesses a calponin-homology domain and IQ repeats, in addition to the RASGAP C-terminal motif (Epp and Chant 1997). MGG_08105 is a homolog of the *S. cerevisiae* GAP *BUD2*, which stimulates hydrolysis of the Ras2 homolog *BUD1*. Mutants defective in *BUD2* display random budding but no obvious growth defect (Park *et al.* 1993). MGG_11425 is a homolog of the *S. cerevisiae* RasGAPs *IRA1* and *IRA2*, which are negative regulators of the Ras-cAMP signaling pathway that is required for reducing cAMP levels under nutrient-limiting conditions (Tanaka *et al.* 1989, 1990).

Ras proteins are low-molecular weight monomeric G-proteins, which localize to the plasma membrane (Wennerberg *et al.* 2005), and switch between the active GTP-bound and inactive GDP-bound status, competitively regulated by GEFs and GAPs (Boguski and McCormick 1993). Ras proteins have intrinsic GTPase and GDP/GTP exchange activity, but GAP and GEF proteins work to ensure a more tightly regulated process. In *S. cerevisiae*, the two Ras proteins, Ras1 and Ras2, are both essential for growth and both function to activate adenylate cyclase (Tamanoi 2011). The RAS/cAMP/PKA pathway in *S. cerevisiae* regulates a variety of processes, including cell cycle progression and life span (Tamanoi 2011). *M. oryzae* also has two Ras-encoding genes, *MoRAS1* and *MoRAS2*, and both have been characterized. In the Δ*ras1* deletion mutant, no distinct phenotypes were observed other than a slight reduction in conidiation (Zhou *et al.* 2014). However, *RAS2* is thought to be an essential gene, and has only been characterized by the generation and expression of a *Ras2* dominant active allele. *MoRAS2^G18V^* transformants formed morphologically abnormal appressoria on both hydrophilic and hydrophobic surfaces, suggesting that dominant active *RAS2* can bypass surface attachment requirements for appressorium formation (Zhou *et al.* 2014). *MoRAS2^G18V^* showed increased Pmk1 phosphorylation and elevated cAMP levels in aerial hyphae. cAMP-PKA signaling has been shown to be important for initial surface recognition and appressorium generation, and for the generation of turgor pressure necessary for infection. The cAMP-dependent PKA mutant, Δ*cpkA*, produces long germ tubes and small nonfunctional appressoria (Xu *et al.* 1997), while the MAP kinase Pmk1 is essential for appressorium formation and invasive growth (Xu and Hamer 1996). In both Δ*pmk1* and Δ*cpka* mutants, expression of *MoRAS2^G18V^* had no effect on appressorium morphogenesis, suggesting that Ras2 functions upstream of both cAMP and Pmk1 signaling pathways (Zhou *et al.* 2014). Deletion of several upstream components of the Pmk1 pathway, including *MST50*, *MST11*, and *MST7*, result in defects in appressorium development and plant infection (Zhao *et al.* 2005; Park *et al.* 2006; Zhou *et al.* 2014). Mst50 functions as an adapter protein of the Mst7-Mst11-Pmk1 cascade involved in activating Pmk1 in *M. oryzae* (Zhao *et al.* 2005). Both Mst50 and Mst11 have been shown to interact with MoRas1 and MoRas2 by yeast two-hybrid assays (Park *et al.* 2006), and deletion of the Ras-association domain of Mst11 blocked Pmk1 activation and appressorium formation (Qi *et al.* 2015), supporting a role for Ras signaling in activation of the Pmk1 pathway. It has been shown recently that the transmembrane mucins Msb2 and Cbp1 function together to recognize extracellular signals through Ras2 (Wang *et al.* 2015). Pmk1 phosphorylation was reduced in a *Momsb2* mutant, but was blocked in a *Momsb2 cbp1* double mutant, which was nonpathogenic (Wang *et al.* 2015). Affinity purification was used to identify a series of Mst50-interacting proteins as well as upstream kinases of the Mps1 pathway, and also the histidine kinase Hik1 (Li *et al.* 2017). These interactions suggest a role for Mst50 in three different signaling pathways. However, domain deletion experiments showed that the Mst50 Ras-association domain was not important for the response to oxidative stress (Li *et al.* 2017). *M. oryzae* Ras2 is therefore essential for cellular viability, and a key mediator between both the Pmk1 MAPK and cAMP signaling cascades (Zhou *et al.* 2014; Qi *et al.* 2015). Activation and deactivation of Ras2 regulates developmental switches/pathways necessary for the growth and pathogenicity of the fungus (Zhou *et al.* 2014).

When considered together, our results suggest that Smo1 acts as a negative regulator of Ras2 and this is why Δ*smo1* mutants display such severe developmental defects, including misshapen spores and appressoria, long germ tubes, and a failure in penetration peg development (see model in Figure 8). These phenotypes point to a defect in the maintenance of polarity that is required for morphological transitions in the fungus. These developmental effects are a consequence of the disruptions to both septin and F-actin dynamics in Δ*smo1* mutants, which are essential for plant infection. *SMO1*-dependent regulation is therefore required for the morphological transitions and cell shape generation processes that are associated with asexual reproduction and plant infection by the blast fungus.
